# Convergence of scaffold-guided bone regeneration and RIA bone grafting for the treatment of a critical-sized bone defect of the femoral shaft

**DOI:** 10.1186/s40001-020-00471-w

**Published:** 2020-12-21

**Authors:** Philipp Kobbe, Markus Laubach, Dietmar W. Hutmacher, Hatem Alabdulrahman, Richard M. Sellei, Frank Hildebrand

**Affiliations:** 1 Department of Orthopaedic Trauma and Reconstructive Surgery, RWTH Aachen University Hospital, Pauwelsstraße 30, 52074 Aachen, Germany; 2 Centre for Regenerative Medicine, Institute of Health and Biomedical Innovation, Queensland University of Technology, Kelvin Grove, QLD Australia; 3 Department of Trauma Surgery and Orthopaedics, Sana Klinikum, Offenbach, Germany

**Keywords:** Scaffold, Polycaprolactone, Tricalcium phosphate, Critical-sized bone defect, Reamer–irrigator–aspirator®

## Abstract

**Background:**

Critical-sized bone defects, mainly from trauma, infection or tumor resection are a challenging condition, often resulting in prolonged, complicated course of treatment. Autografts are considered as the gold standard to replace lost bone. However, limited amount of bone graft volume and donor-site morbidity have established the need for the development of alternative methods such as scaffold-based tissue engineering (TE). The emerging market of additive manufacturing (3D-printing) has markedly influenced the manufacturing of scaffolds out of a variety of biodegradable materials. Particularly medical-grade polycaprolactone and tricalcium phosphate (mPCL–TCP) scaffolds show appropriate biocompatibility and osteoconduction with good biomechanical strength in large preclinical animal models. This case report aims to show first evidence of the feasibility, safety, and efficacy of mPCL–TCP scaffolds applied in a patient with a long bone segmental defect.

**Case presentation:**

The presented case comprises a 29-year-old patient who has suffered a left-sided II° open femoral shaft fracture. After initial external fixation and subsequent conversion to reamed antegrade femoral nailing, the patient presented with an infection in the area of the formerly open fracture. Multiple revision surgeries followed to eradicate microbial colonization and attempt to achieve bone healing. However, 18 months after the index event, still insufficient diaphyseal bone formation was observed with circumferential bony defect measuring 6 cm at the medial and 11 cm at the lateral aspect of the femur. Therefore, the patient received a patient-specific mPCL–TCP scaffold, fitting the exact anatomical defect and the inserted nail, combined with autologous bone graft (ABG) harvested with the Reamer–Irrigator–Aspirator system (RIA—Synthes®) as well as bone morphogenetic protein-2 (BMP-2). Radiographic follow-up 12 months after implantation of the TE scaffold shows advanced bony fusion and bone formation inside and outside the fully interconnected scaffold architecture.

**Conclusion:**

This case report shows a promising translation of scaffold-based TE from bench to bedside. Preliminary evidence indicates that the use of medical-grade scaffolds is safe and has the potential to improve bone healing. Further, its synergistic effects when combined with ABG and BMP-2 show the potential of mPCL–TCP scaffolds to support new bone formation in segmental long bone defects.

## Case report

### Background

Critical-sized bone defects (> 5 cm) can be the result of a traumatic event, a malignant disease or (infectious) non-union. Currently established treatment options for the reconstruction of such large bone defects are autologous (vascularized) bone transfer, segmental bone transport or the Masquelet technique.

Yet, all of the above listed techniques have several drawbacks. Autologous bone transfer not only possesses a substantial harvesting morbidity, but also the exact fitting of the graft into the defect zone is often demanding and bony ingrowth of the graft is frequently not achieved right away. Segmental transport is a very lengthy and demanding procedure and often associated with external stabilization and therefore with low patient comfort. Further, the final docking process of the transported bone often requires several docking procedures [1, 2]. Standalone Masquelet technique has shown promising results in some surgeons practice; however graft resorption appears to be a major problem with highly heterogeneous bone formation capacity often resulting in multiple additional interventions [3]. Further, it remains undetermined which osteoinductive and osteoconductive material should best be put inside the Masquelet membrane [4, 5].

In recent years, significant advances have been made in the convergence of 3D-printing and scaffold-guided bone regeneration [6–8]. For example, biodegradable, customized composite scaffolds can be 3D-printed by using medical-grade polycaprolactone (mPCL) in combination with β-tricalcium phosphate (β-TCP), which can be precisely fitted into the bony defect zone.

### Case presentation

**Fig. 1:**
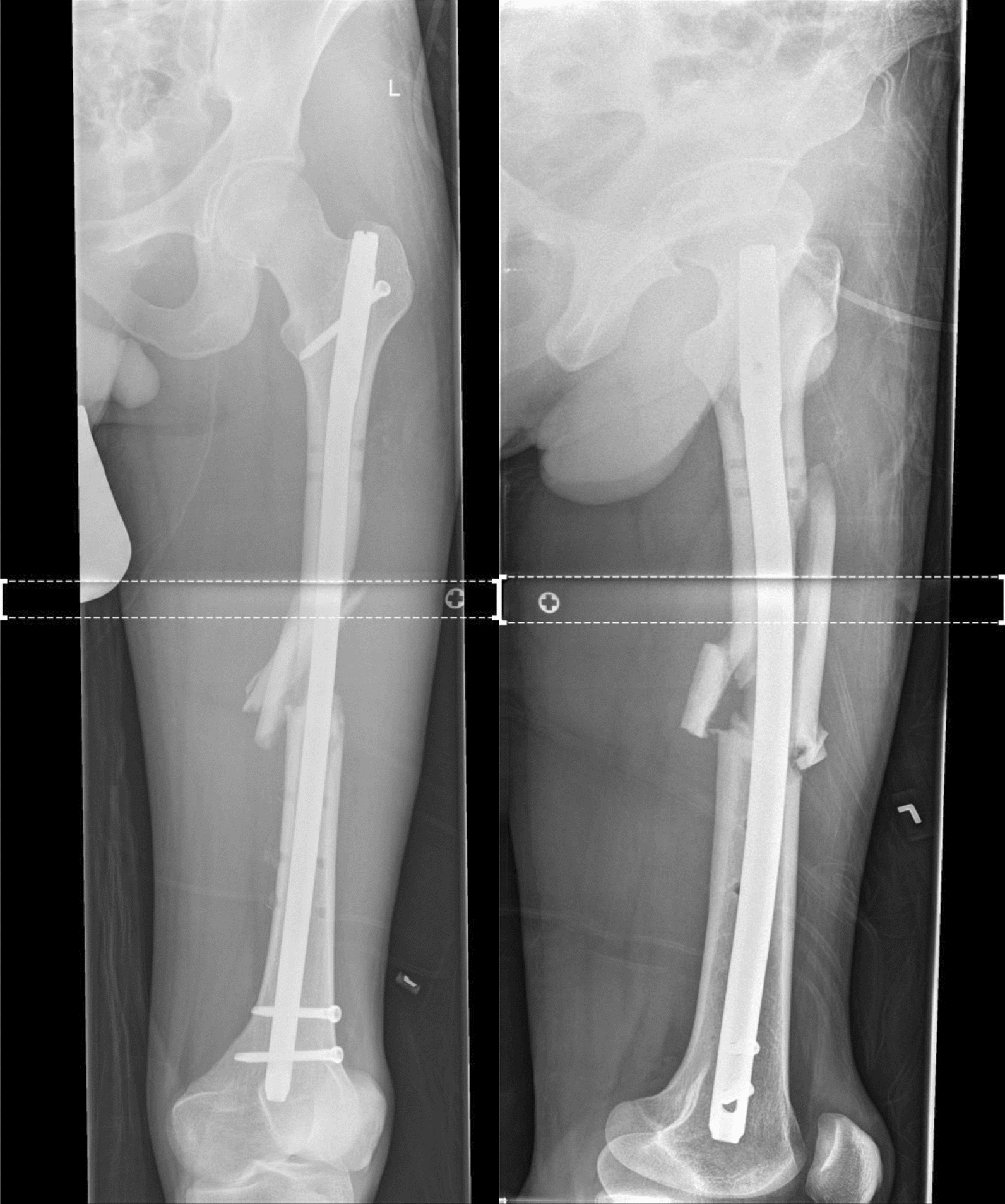
Left-sided II° open femoral shaft stabilized with a reamed antegrade femoral nail

**Fig. 2:**
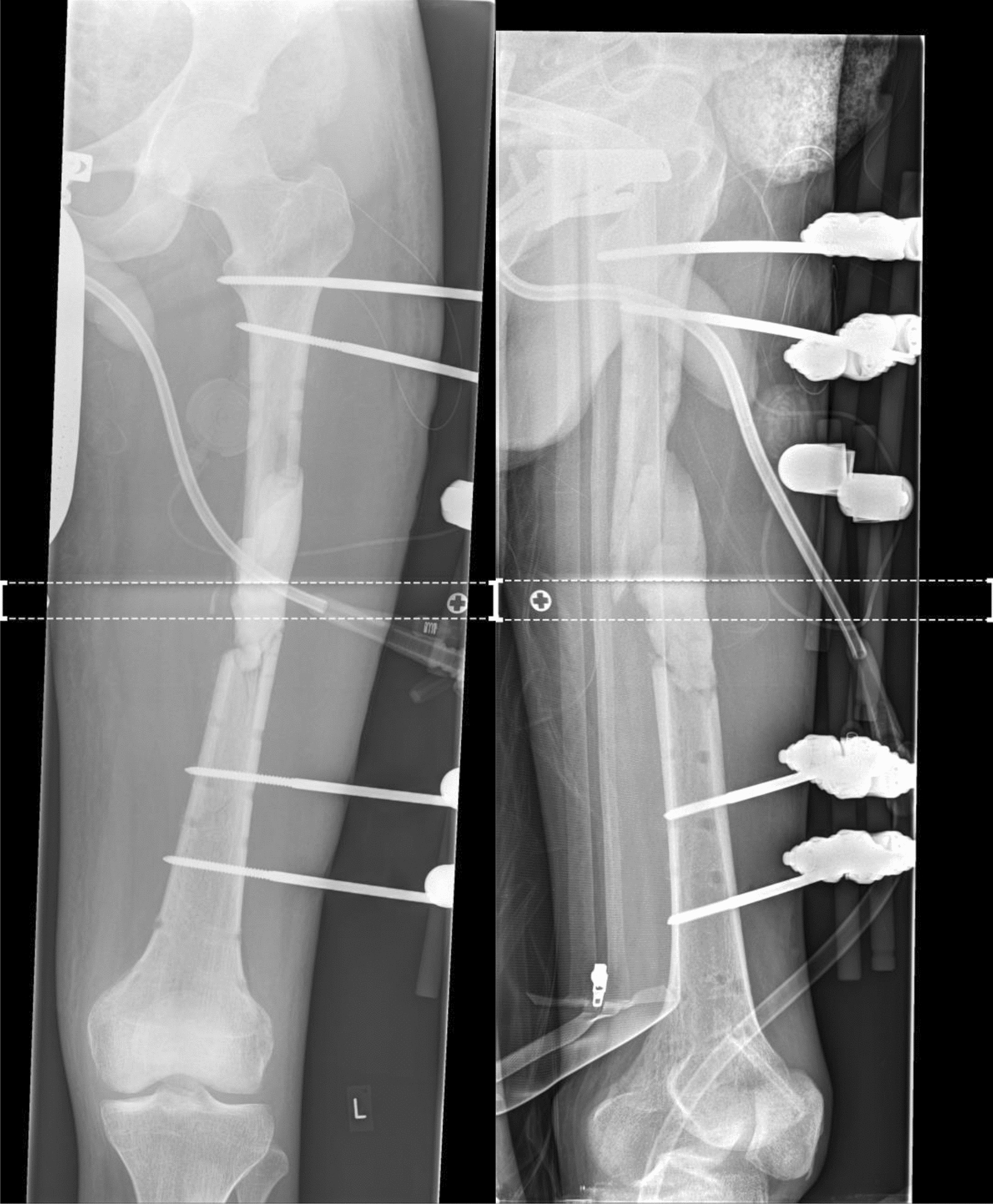
Preparation of the defect for Masquelet technique by insertion of a vancomycin cement seal in order to induce a vascularized membrane

**Fig. 3:**
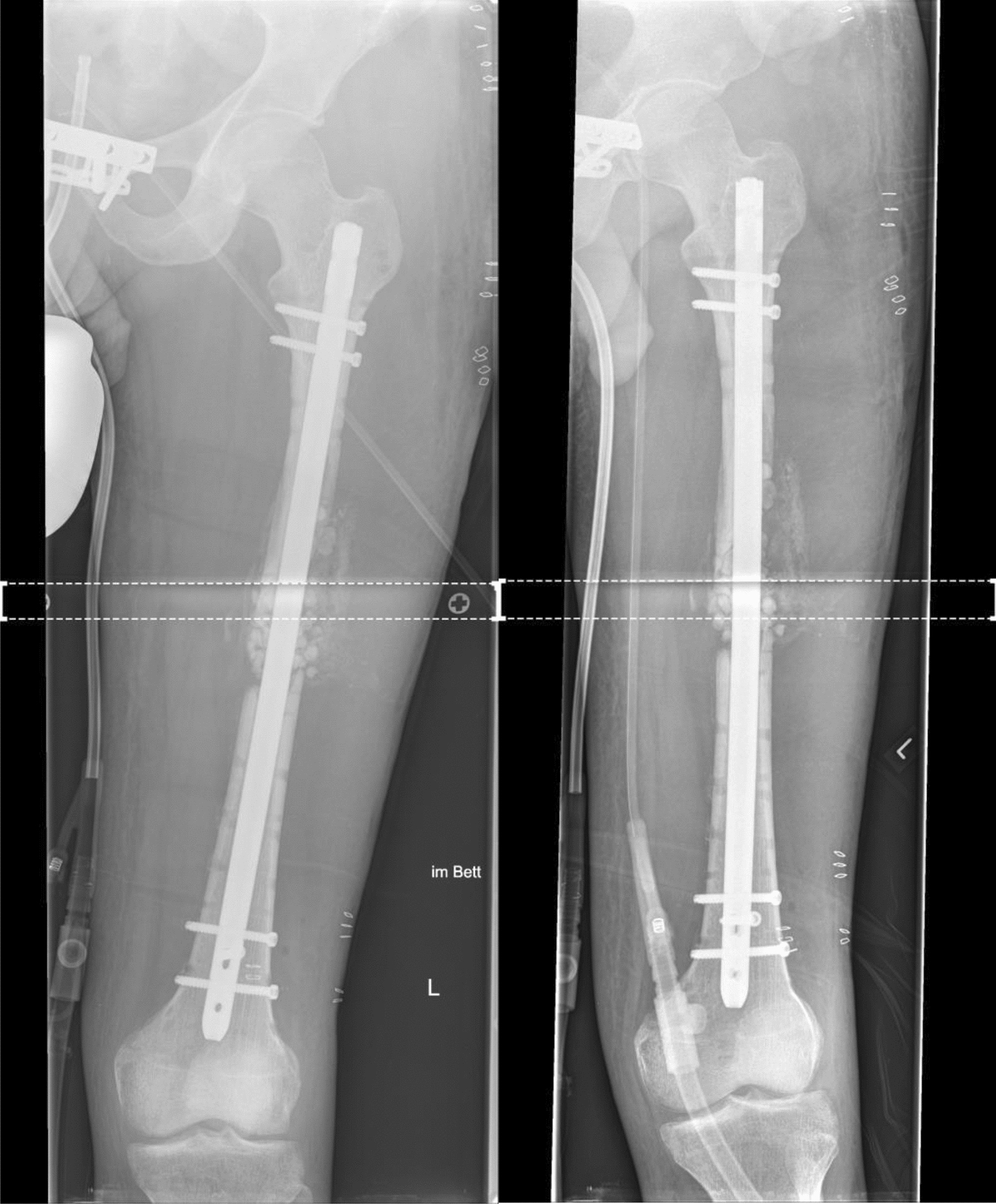
Reamed antegrade femoral nail and Masquelet membrane filled RIA bone grafting mixed with Cerament G®

A 29-year-old patient suffered a left-sided II° open femoral shaft fracture and an unstable pelvic injury. Initially, the bony injuries were stabilized with external fixation and converted to a definitive stabilization with a reamed antegrade femoral nail for the femoral shaft fracture after one week (Fig. 1). Six weeks following the nail insertion, an infection in the area of the formerly open fracture with a multi-resistant Gram-negative Escherichia coli (3-MRGN) occurred, requiring nail removal, resection of avital bone, subfascial vacuum therapy and external fracture fixation. During subsequent revision surgeries through a lateral approach to the femur, sterile wound conditions were achieved, and the bony defect prepared for Masquelet technique by insertion of a vancomycin cement seal in order to induce a vascularized membrane (Fig. 2). Four weeks after induction of the Masquelet membrane, the vascularized membrane was cautiously opened through the lateral approach and the cement seal was removed. Now, the external fixation was removed, and the femoral defect was again bridged and stabilized with a reamed antegrade femoral nail, which was cautiously passed through the former position of the cement seal surrounded by the vascularized membrane. The membrane was then filled with Reamer–Irrigator–Aspirator® (RIA) bone grafting, harvested from the contralateral femur, mixed with Cerament G® (BONESUPPORT AB, Lund, Sweden) and finally closed by suture (Fig. 3). The microbiological samples obtained during this operation again confirmed sterile wound conditions in long-term incubation.

**Fig. 4:**
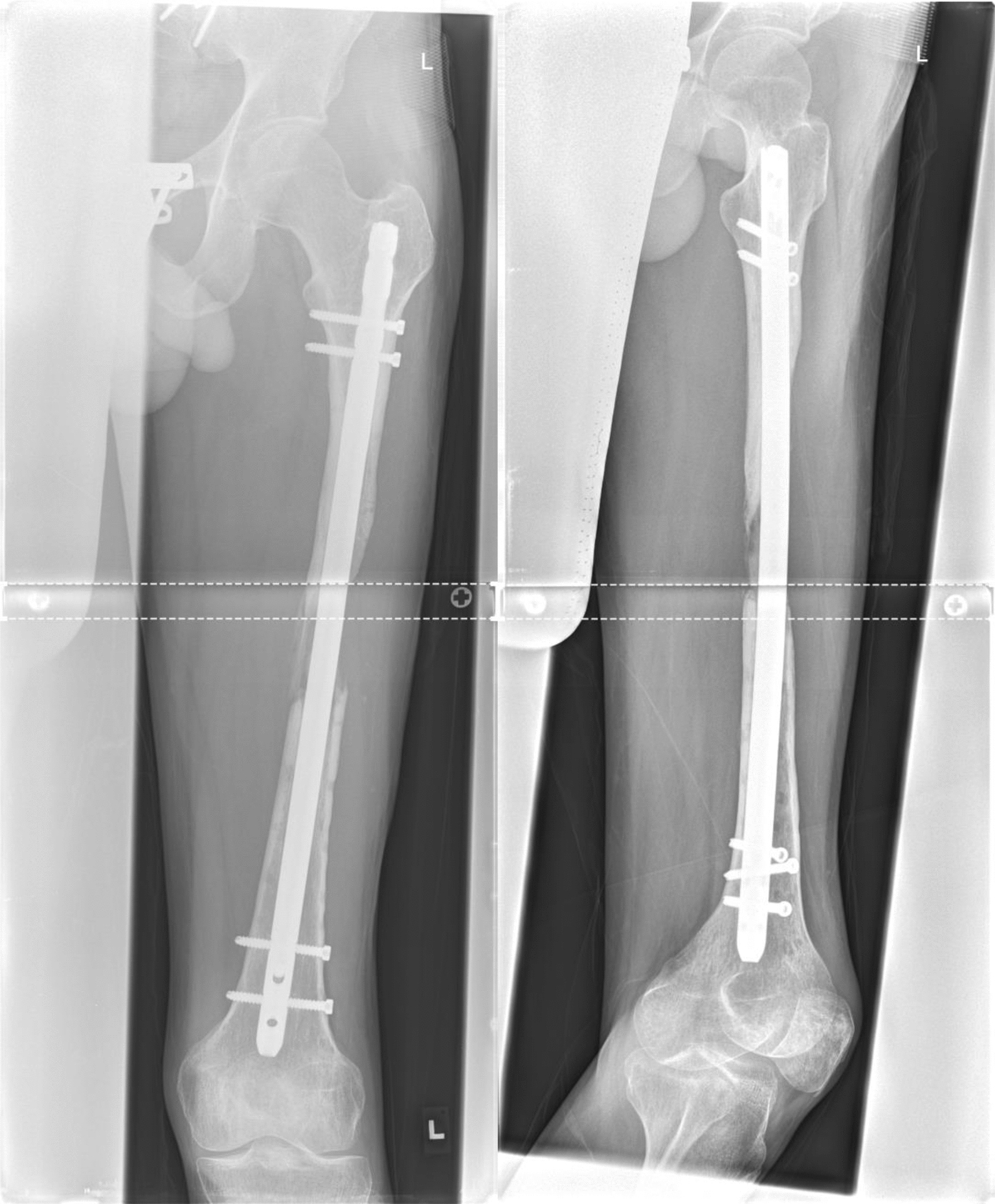
RIA bone graft resorption and persistent non-union 10 months following surgery

**Fig. 5:**
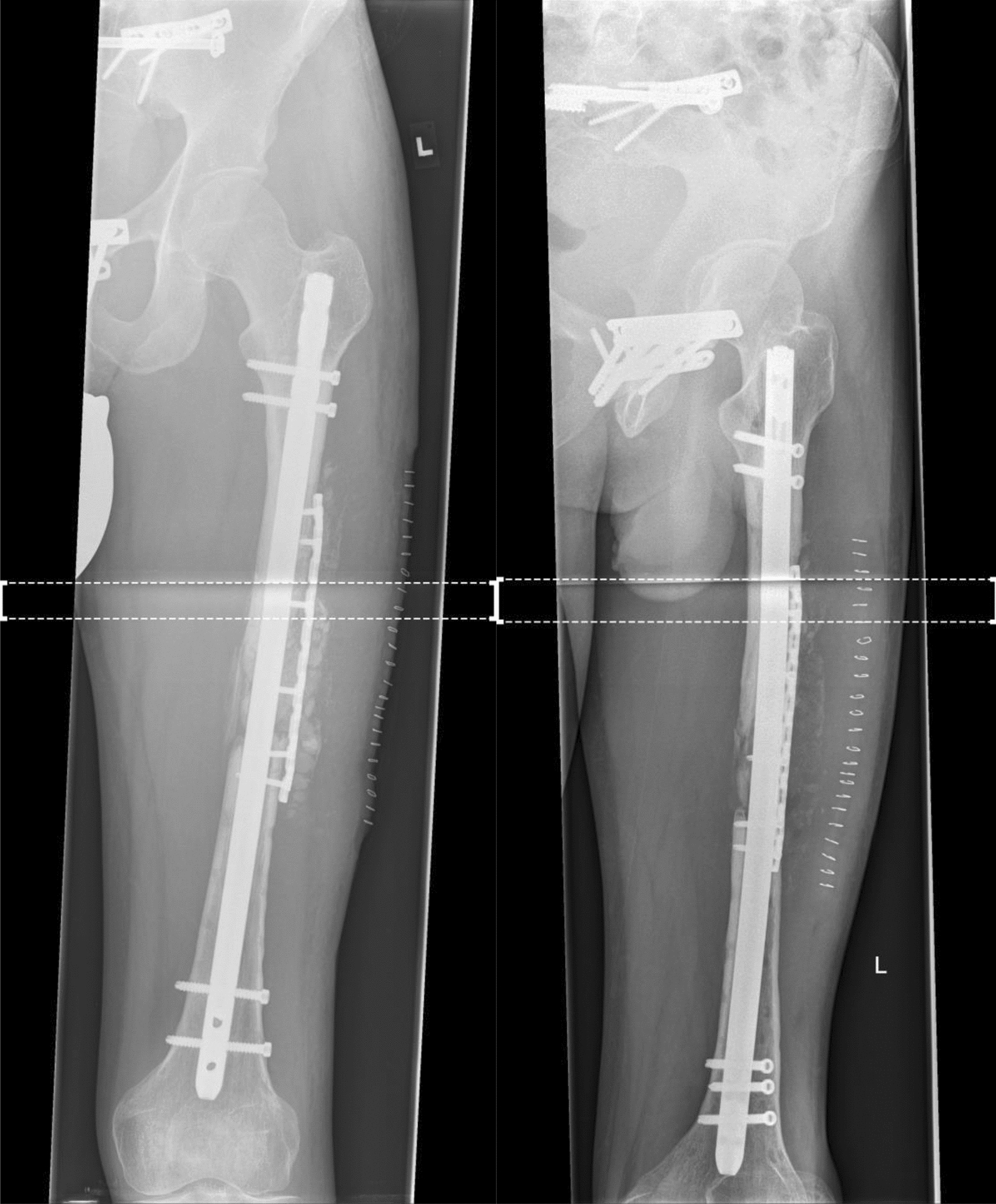
Revision surgery with tricortical iliac crest graft, Cerament G® and additionally plating with a reconstruction plate

Ten months following the antegrade nail insertion and the Masquelet grafting procedure, the patient presented with ongoing pain during loadbearing and radiologically showed a persistent non-union (Fig. 4). Within revision surgery, non-vital bone was resected and a tricortical iliac crest graft inserted press fit in the lateral aspect of the femur and additionally secured with a reconstruction plate (Fig. 5). Additionally, Cerament G® was applied to the lateral aspect of the non-union.

**Fig. 6:**
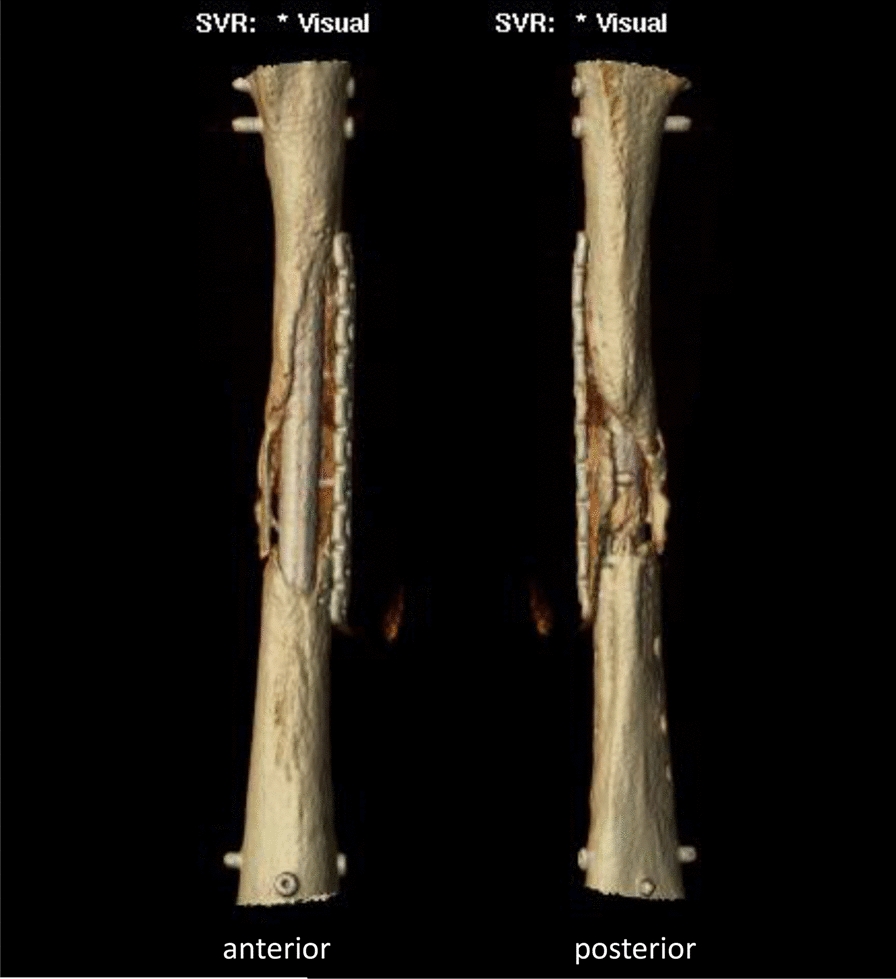
Completely resolved tricortical iliac graft 7 months after surgery

Seven months after tricortical iliac crest grafting, the patient suffered persistent pain during loadbearing and radiologically the graft was almost completely resolved (Fig. 6). Due to the failure of achieving bone healing applying commonly used methods the patient was then prepared for insertion of a customized, biodegradable 3D mPCL–TCP scaffold (Table 1).

**Table 1:** Summary of surgical methods applied to achieve bone healing prior to utilization of an individual healing attempt with a tissue-engineered construct including a medical-grade polycaprolactone and tricalcium phosphate scaffold

Time after trauma	Diagnosis	Surgical treatment
+ 6 weeks	Local infection with multi-resistant Gram-negative Escherichia coli with intramedullary nail (IMN) in situ	Procedural change to external fixation and Masquelet technique with vancomycin-loaded cement spacer
+ 10 weeks	Critical-size femoral defect with established Masquelet induced membrane	IMN fixation and Reamer–Irrigator–Aspirator® (RIA) bone grafting mixed with Cerament G®
+ 14 months	Persistent pain and non-union	Insertion tricortical iliac crest graft (lateral aspect of femur) with Cerament G® and secured with additional plate
+ 21 months	Resorption of tricortical iliac crest graft and ongoing pain during loadbearing	Implantation of patient-specific medical-grade scaffold in combination with autologous bone graft and bone morphogenetic protein-2

**Fig. 7:**
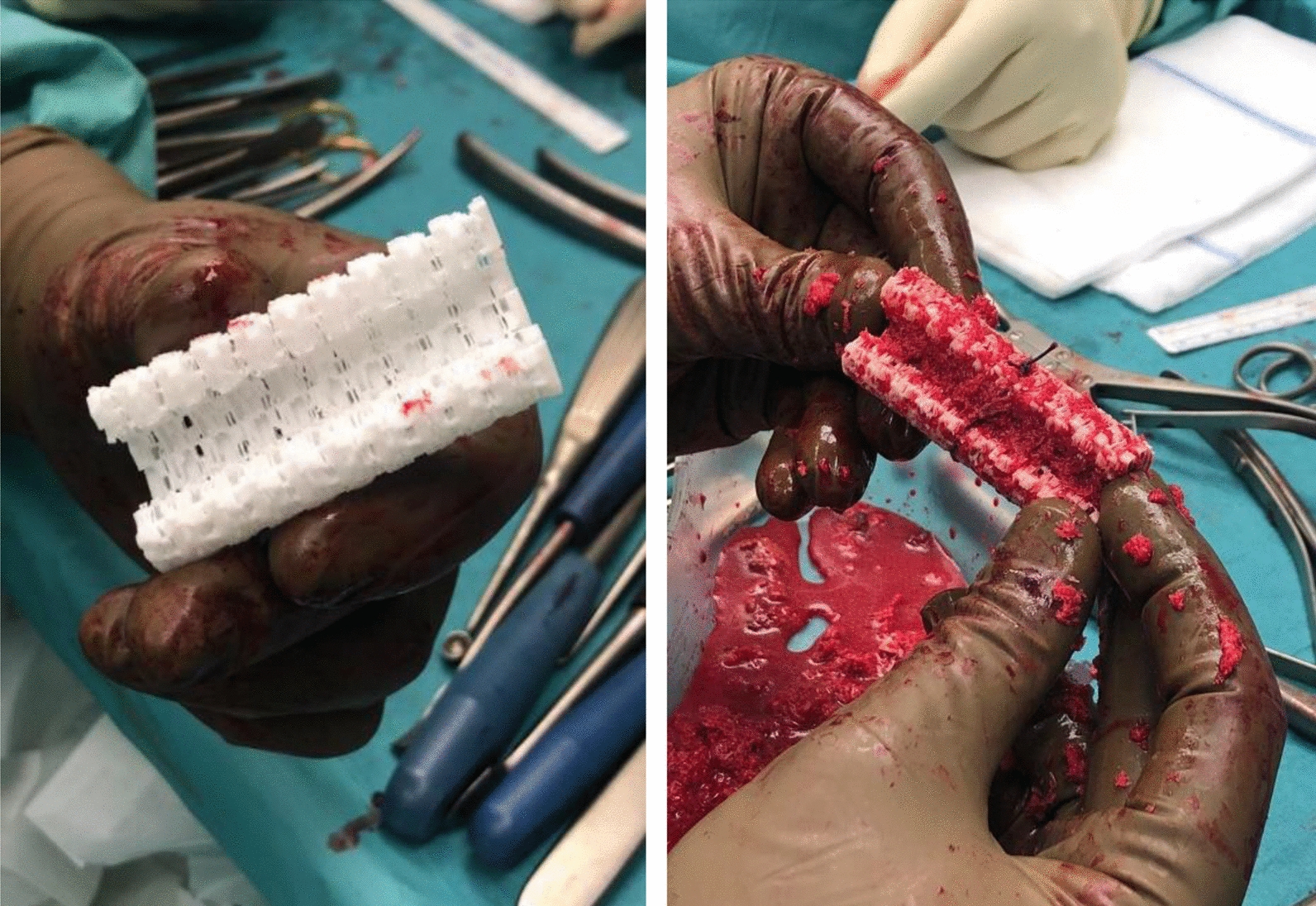
Customized, biodegradable 3D mPCL–TCP scaffold (Osteopore®) filled with RIA bone grafting

**Fig. 8:**
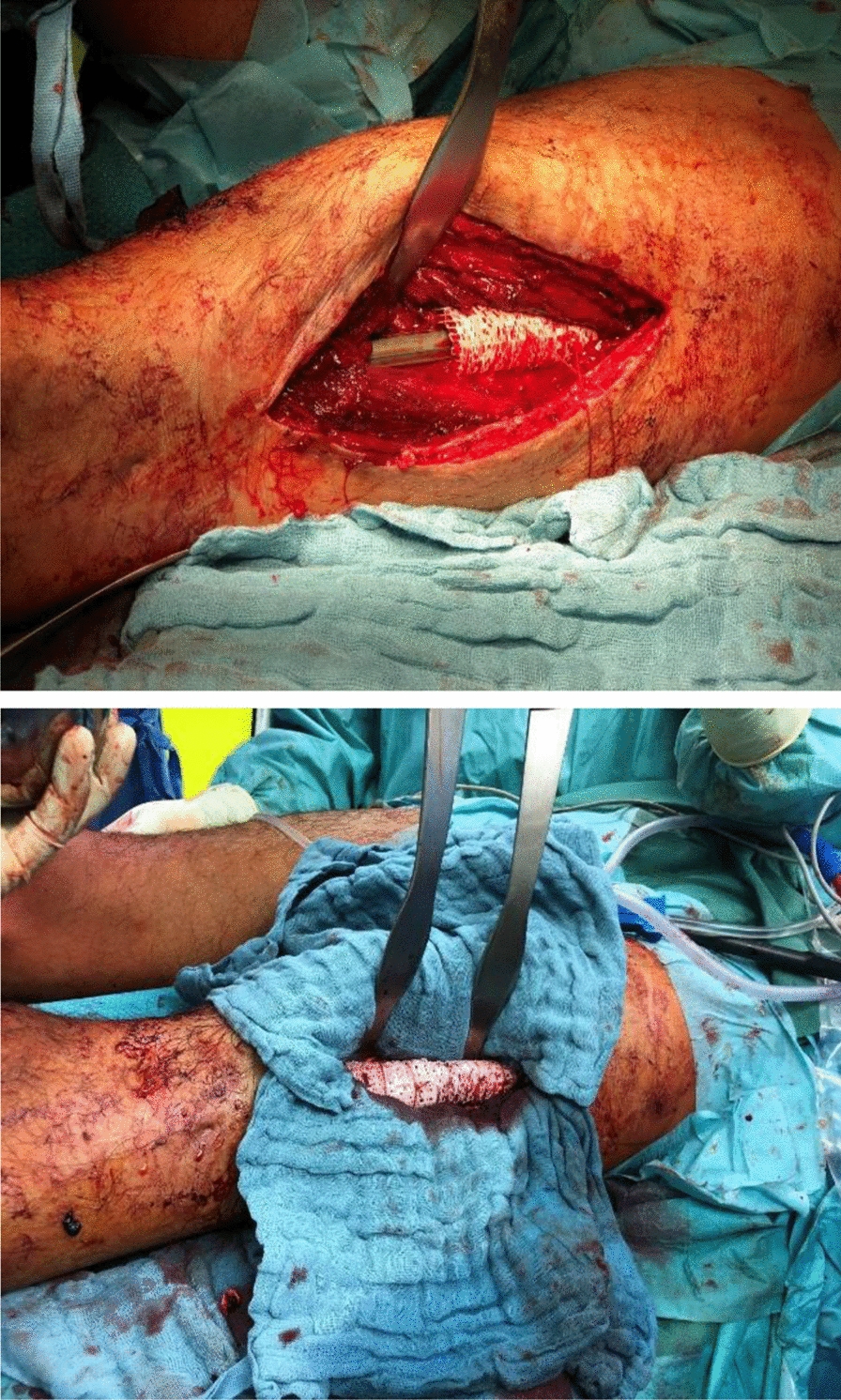
The scaffold was circumferentially pressed on the nail and finally covered with a BMP-2-impregnated membrane

Therefore, a computed tomography (CT)-scan was obtained and processed by Osteopore® to produce an individualized scaffold by additive manufacturing. The circumferential bony defect now measured 6 cm at the medial and 11 cm at the lateral aspect of the femur. During surgery, the reconstruction plate was removed, and the nail was circumferentially exposed over the former lateral access. RIA bone grafting was obtained from the ipsilateral tibia and filled into the customized mPCL–TCP scaffold (Fig. 7). Then, the three composite scaffolds were circumferentially pressed on the nail to fill the defect space (Fig. 8) and finally covered with a bone morphogenetic protein-2-impregnated collagen membrane.

**Fig. 9:**
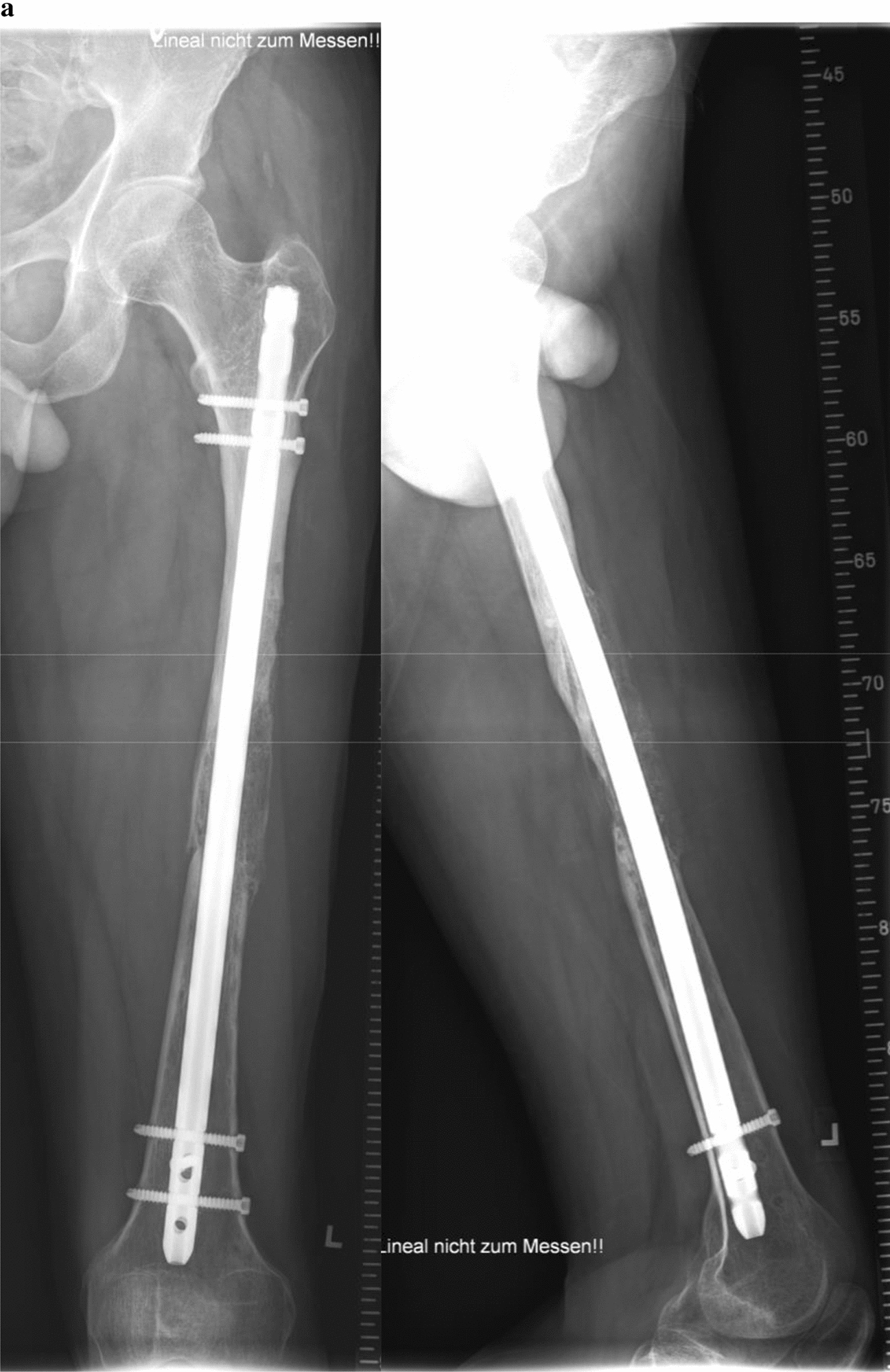

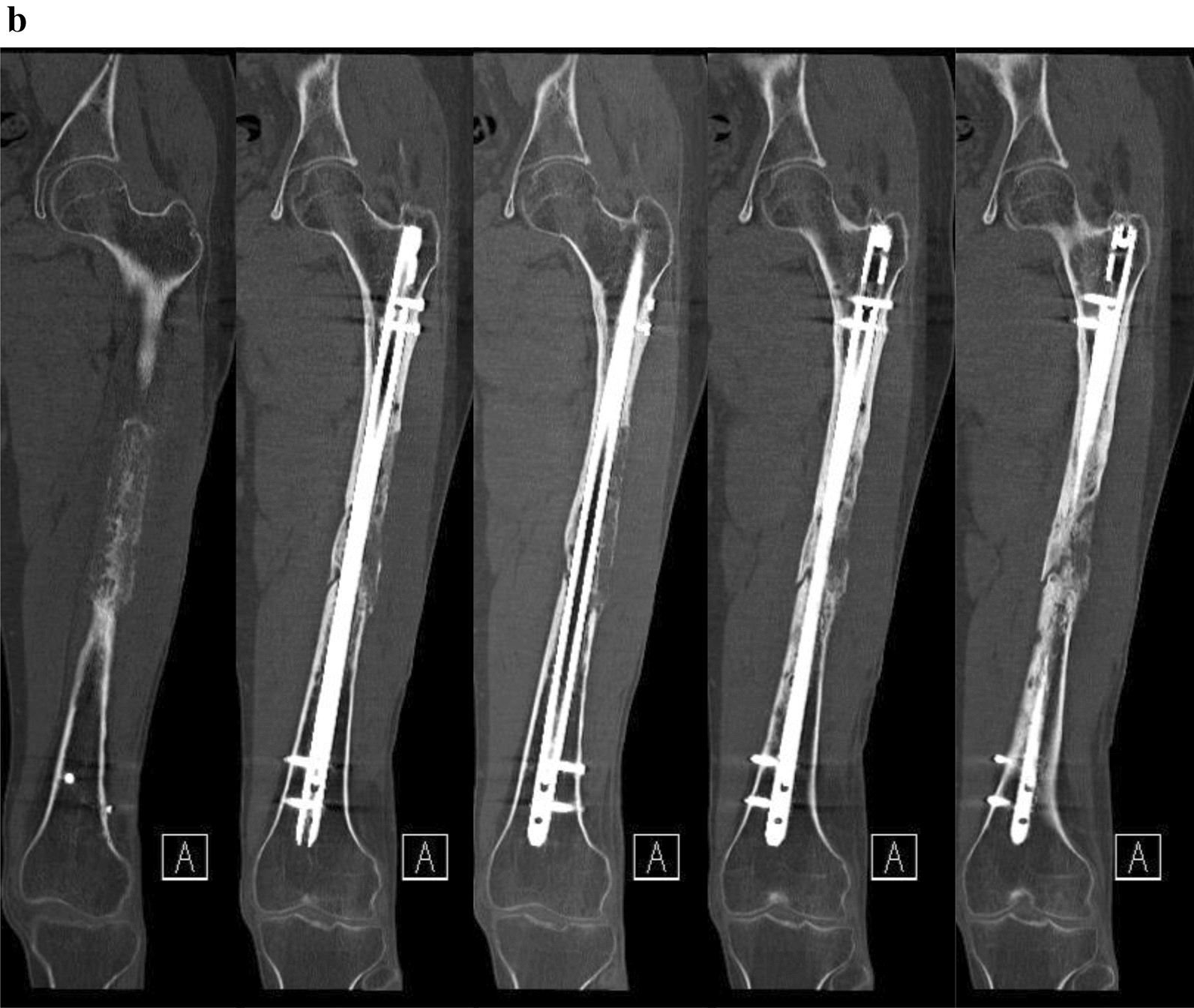
**a** X-ray 12 months after surgery shows delicate but adequate bone formation. **b** CT-scan 12 months after surgery confirms almost complete bony fusion of the critical-sized defect with partial degradation of the mPCL–TCP scaffold

Twelve months after surgery, the patient presented with no pain under full weight bearing and the X-ray shows delicate but adequate bone formation. A CT-scan confirms almost complete bony fusion of the critical-sized defect and bone formation inside and outside the fully interconnected scaffold architecture (Fig. 9).

## Discussion

A paramount requirement for healing of critical-sized defects is the establishment of an osteogenetically inductive and conductive environment paired with mechanical stability (diamond concept) [9]. Autologous grafts, like vascularized fibula transfer or iliac crest, offer a satisfying combination of above-mentioned biological and mechanical properties and are therefore still considered the gold standard [10]. However, its use is limited by several factors, including limited bone graft size and volume and donor-site morbidity with persistent pain at the iliac crest after bone graft harvesting in up 30% [11–13]. Further, critical-sized defects filled with a fibula or iliac crest graft are usually stabilized either with a plate or an external fixation because they are not accessible for a biomechanically superior nail osteosynthesis due to a missing canal. Nail osteosynthesis may be performed in combination with the Masquelet technique [14]: after induction of the Masquelet membrane the void is filled with RIA bone grafting. Despite several reported excellent results in the literature as well as in our hands [4, 15, 16], extensive graft resorption and weakness of the reconstructed segment, probably due to a missing osteoconduction, is a major drawback of this technique. The problem of RIA bone graft resorption despite insertion into a vascularized membrane can be seen in the above illustrated case. The failure of the tricortical iliac crest graft in our presented case may be due to two factors: first, this type of graft is difficult to insert press fit into a larger three-dimensional complex defect, especially in case of an already inserted nail, and secondly only partly addresses the volume of the circumferential defect.

In the past decades, scaffold-guided bone tissue engineering has emerged as a promising strategy to overcome the shortcomings associated with established techniques [17–19]. The ability of 3D-printing allows the design and manufacture of osteoconductive scaffolds which are optimized for clinical translation in terms of pore size, layering, and degradation [20]. Equipping the scaffolds with osteogenic as well as osteoinductive properties is a condition sine qua non; yet this is a highly demanding process with several challenges. For example, the seeding of the scaffold with mesenchymal precursor cells in order to gain osteogenetic properties is possible; however, several drawbacks have to be kept in mind. First, this requires a harvesting surgical procedure and an ex vivo cultivation of the cells, which has been shown to reduce the osteogenetic potential as well as affect phenotype and behavior of these cells [21, 22]. Secondly, sterilization of the seeded scaffolds is difficult and may further reduce the biological potential. Thirdly, new biodegradable material directly coupled with a biologic may face the most difficult FDA class 3 regulatory approval [23]. So overall, this is an extremely demanding and expensive process reducing the suitability for routine clinical use.

Our approach, illustrated in the above presented case, separates the diamond concept into three independent workflows, which are easily merged during surgery: 3D-printing of a well-designed biodegradable scaffold with osteoconductive properties, which is intraoperatively packed with osteogenetic and osteoinductive highly potent RIA bone grafting [24–26]. Mechanical stability is achieved with an intramedullary nail on which the customized RIA bone graft filled scaffold is circumferentially clipped.

There are various advantages of this clinically driven methodology. First of all, this approach allows usage of an intramedullary nail as the mechanically most robust implant for long bone stabilization with critical-sized defects. Secondly, customized printing according to a CT-scan allows for an individualized and optimal fit of the scaffold in the defect and around the nail. Further, the 3D-printing in layering technique allows creation of a high porosity (70%) with interconnected pores of 800–2000 µm, which is reported to be a design requirement for large-volume segmental tibia and femur defect in a preclinical model [27, 28]. The usage of medical-grade PCL and ß-TCP in an 80:20 ratio further offers the suitable mechanical properties and degradation kinetics by hydrolysis as compared to unpredictable resorption of fast degrading natural and synthetic polymers. Briefly, PCL is a biopolymer with excellent biocompatibility and biodegradability [29] causing no local inflammation [30] and no accumulation in organs [31]. The pore geometry of a scaffold with collagen fiber network eventually functions as a cell-deposit template. Thereby, it fosters vascular ingrowth which builds a proper microenvironment facilitating oxygen and nutrient transport to the inner part of the scaffold essential for bone repair and crucial for avoiding premature bone graft resorption [18, 32]. By inclusion of TCP and manufacturing of the mPCL–TCP composites osteoconductivity of scaffolds further increases resulting in the production of a scaffold providing structural support for cell attachment and tissue development suitable for clinical application in combination with autologous bone grafting [6, 33].

Thus, the intraoperative packing of the mPCL–TCP scaffold with RIA bone grafting adds excellent osteogenic as well as osteoinductive properties without ex vivo cultivation and minimal reported donor-site morbidity [34].

## Conclusion

Healing of critical-sized bone defects remains a challenge for the orthopedic surgeon. Despite a better understanding of the difference between fracture healing and non-unions/large-volume bone defect regeneration in recent years, the biological and mechanical requirements for healing remain unchanged. We described a pragmatic and easy approach separating the fundamental pillars of non-unions in three different workflows combined within the surgery making this approach likely a candidate for future routine clinical application.

## Data Availability

All data generated or analyzed during this study are included in this published article.
